# Augmentation-Mastopexy With 4-Layer Autologous Fat Grafting and Evaluation of Viability With MRI

**DOI:** 10.1093/asjof/ojae046

**Published:** 2024-06-24

**Authors:** Hüseyin Kandulu

## Abstract

**Background:**

Autologous fat (AF) grafting is widely used in plastic surgery and is generally considered a safe and effective procedure. A combined approach utilizing vibration amplification of sound energy at resonance (VASER) to prepare AF grafts with a 4-layer fat grafting technique was explored in this study.

**Objectives:**

To offer a customized solution that accommodates individual anatomical differences.

**Methods:**

This retrospective, cross-sectional case series involved 40 breasts from 20 female patients who underwent primary augmentation-mastopexy. After removing excess breast tissue and exposing the pedicle, lateral pillars, and pectoral muscle, the 4-layer fat grafting was performed as follows: 150 mL of AF under the pectoral muscle directed toward the medial and central zones; 100 mL into the pectoral muscle toward these zones; 50 mL under the pectoral fascia, moving retrograde from cephalic to caudal portions at a 30° to 45° cannula angle. After closing the epithelial and subepithelial incisions, an additional 100 to 150 mL of AF was injected under the subcutaneous layer around the breast, depending on each patient's contour and breast shape. Patients were monitored for 2 years with MRI scans to assess breast volume, anatomy, and fat graft survival.

**Results:**

The average follow-up was 26 ± 2.81 months. MRI evaluations indicated an efficient survival rate of the fat grafts. None of the patients experienced minor or major complications.

**Conclusions:**

The 4-layer AF grafting technique appears to be a safe and effective procedure for customized breast sculpting in augmentation-mastopexy surgery, with a high rate of fat graft sustainability and survival.

**Level of Evidence: 4:**

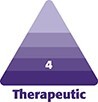

According to the International Society of Aesthetic Plastic Surgery International Survey on Aesthetic/Cosmetic Procedures in 2021, breast augmentation was the most common surgical procedure for females, alongside breast lifts, which increased by 31.4% compared with the previous year.^1^

Although the use of surgical implants is common, a prosthetic reconstruction of the breast might cause complications including displacement, infection, pain, capsular contracture, inadequate coverage, and asymmetry. Moreover, breast implants are associated with an increased revision rate within the first decade after the surgery due to these complications or various individual causes.^2^

Autologous fat (AF) grafting is an easy and efficient method for the harvesting of even smaller amounts of adipose tissue and is related to a less incidence of donor-site complications.^3^

Despite the previous concerns regarding its interference with cancer detection and diagnostics, breast augmentation by AF grafting was accepted by the Fat Graft Task Force of the American Society of Plastic Surgeons as a safe procedure in 2009 with the improvements in radiological imaging technologies, identifying the AF grafts, microcalcifications, and suspicious lesions.^4^

With the accumulation of data and experience worldwide for breast reconstruction using AF grafts, the procedure gains popularity for the augmentation, reconstruction, and contouring of breast tissue. In addition, it allows the correction of soft-tissue deformities, and a tailored shaping of individual breasts to improve the shape variations and fullness of the upper and medial poles.^5^

Vibration Amplification of Sound Energy at Resonance (VASER) is an ultrasound-assisted technology related to a lower incidence of damage to the surrounding tissues and adipose tissue. The evidence suggests an increased adipocyte and stromal tissue survival with the use of VASER technology for a liposuction procedure, providing a more viable fat harvesting process.^6,7^

Although the hybrid breast augmentation with implants and AF transfer has been evaluated in several studies, the most appropriate locations receiving the grafts, and the harvesting and preparation methods in order to provide natural looking and enduring breast anatomy have been examined in a limited number of studies.^8,9^ Thus, we aim to describe a 4-layer AF grafting method for patients who underwent a primary augmentation-mastopexy procedure, using the grafts harvested with VASER assistance, and present its 2-year follow-up outcomes confirmed by MRI studies.

## METHODS

In this retrospective, cross-sectional, MRI imaging enrolled study, we enrolled 40 breasts of 20 female patients who underwent a primary augmentation-mastopexy procedure between June 2020 and December 2023. All patients provided informed consent, and the study was conducted in accordance with the Declaration of Helsinki. All operations were performed at a single clinic by the same surgeon. The inclusion criteria were: female patients >18 years, requiring augmentation-mastopexy surgery, a negative preoperative mammogram, and adequate adipose tissue for harvesting. Patients with congenital defects of the chest and breast area, previous individual or family history of breast cancer, reluctance for an MRI scan, and who required a prosthetic implant were excluded. In addition, patients with contraindications and comorbidities for liposuction and mastopexy surgery, a history of previous liposuction, and breast surgery were ruled out. All cases were asked to stop smoking at least 2 weeks before and 2 weeks after the procedures. The following demographic, clinical, and technical data were collected: age, BMI, smoking status, parity, type of breast incision, the quantity of fat harvested, quantity of fat reinjected per breast, and postoperative complications. Anterior and lateral photographs were taken before the operation, the regions of interest were marked with different color markers in an upright standing position, and the degree of ptosis and preoperative measurements were recorded. All patients underwent a conventional mastopexy procedure using either a vertical or an inverted-T scar method, and the AF grafting was performed on different and individually determined parts of the breast tissue depending on the requests and anatomic requirements of the patient. The AF grafts were obtained with a VASER (Solta Medical, Hayward, CA)-assisted liposuction procedure, and the viability, distribution, and stability of the inoculated fat tissue were evaluated using an MRI scan on the second year follow-up visits.

### Liposuction and Fat Harvesting Procedure

All patients received intravenous antibiotics prior to surgery and were operated on under general anesthesia. After surgical preparation with povidone-iodine solution, the region of the patient for harvesting was covered with a sterile drape. A tumescent solution prepared with 1 mg adrenaline and 10% lidocaine hydrochloride in each 1000 mg Ringer's lactate solution was infiltrated in the areas of liposuction and fat harvesting. VASER-assisted liposuction was performed by the conventional technique using 16-gauge multiple-hole infiltration cannulas (1.2 mm of inner diameter) and 50 mL syringes on selected regions, with the VASER mode set to 100% C.

### Preparation and Injection of the Adipose Tissue Grafts

Harvested adipose tissue grafts were obtained from the lower abdominal area in all patients. The lipoaspirates collected into an air-proof system were kept in an upright position, allowing the separation of fat and fluid portions by the effect of gravity. The adipose tissue transfers were made with the patient in a supine position. After the excision of excess breast tissue and exposure of pedicle, lateral pillars, and pectoral muscle, the 4-layer fat grafting was applied as follows: an AF of 150 mL under the pectoral muscle toward the medial and central zones; 100 mL into the pectoral muscle toward the medial and central zones; 50 mL under the pectoral fascia in a retrograde manner from cephalic to caudal portions, at a cannula angle of 30° to 45°. Following the closure of epithelial and subepithelial incisions, a total of 100 to 150 mL AF was given under the subcutaneous layer surrounding the breast with microinjections, depending on the individual contour and shape of the breast for each patient (Video).

Injections were administered beneath and within the muscle tissue using 50 cc syringes and cannulas with a diameter of 3 to 2 mm. For subcutaneous injections, 20 cc syringes and cannulas with a diameter of 1.8 to 1.4 mm were used.

Patients were followed up at the end of 18 months with MRI scans for the evaluation of breast volume, anatomy, and the survival of the AF graft, using a 1.5 T MRI scanner (Siemens Healthineers, Erlangen, Germany), and multiple T_1_-weighted sequences were obtained. All MRI scans were read by the same radiologist.

### Statistical Analysis

Data were analyzed using the GraphPad Prism version 8.0.0 for Windows (GraphPad Software, San Diego, CA). The data were presented as mean ± standard deviation (range).

## RESULTS

The study group consisted of 40 breasts of 20 female patients with an average age of 35.88 ± 6.05 years (range, 25-41 years) with a mean BMI of 25.64 ± 2.77 kg/m^2^ (range, 20.34-32.4 kg/m^2^)

Demographical and clinical data of the patients are presented in Table 1.

**Table 1.** Demographic and Clinical Variables of the Patients (*n* = 20)

Characteristic	Mean ± SD (#, %)	Range
Age (years)	35.88 ± 6.05	25-41
BMI (kg/m^2^)	25.64 ± 2.77	20.34-32.4
Smoking		
No	14 (70)	
Yes	6 (30)	
Parity		
0	10 (50)	
1	5 (25)	
2	4 (20)	
4	1 (5)	
Lipoaspirate volume (mL)	1213 ± 825	600-3200
Injected fat graft volume, left (mL)	424 ± 19.65	400-450
Injected fat graft volume, right (mL)	425 ± 19.46	400-450
Follow-up duration (months)	26 ± 2.81	24-31

SD, standard deviation.

Seven patients underwent the primary augmentation-mastopexy procedure with a vertical incision, whereas an inverted-T incision procedure was employed for 13 individuals

Total fat injected per breast ranged from 400 to 450 mL in a single session

The total duration of the operation ranged from 108 to 120 min (mean 112 min)

The average follow-up was 26 ± 2.81 months (range, 24-31 months)

None of the patients developed cystic masses, calcifications, infection, or complications at the recipient and/or donor sites

**Figure 1.**
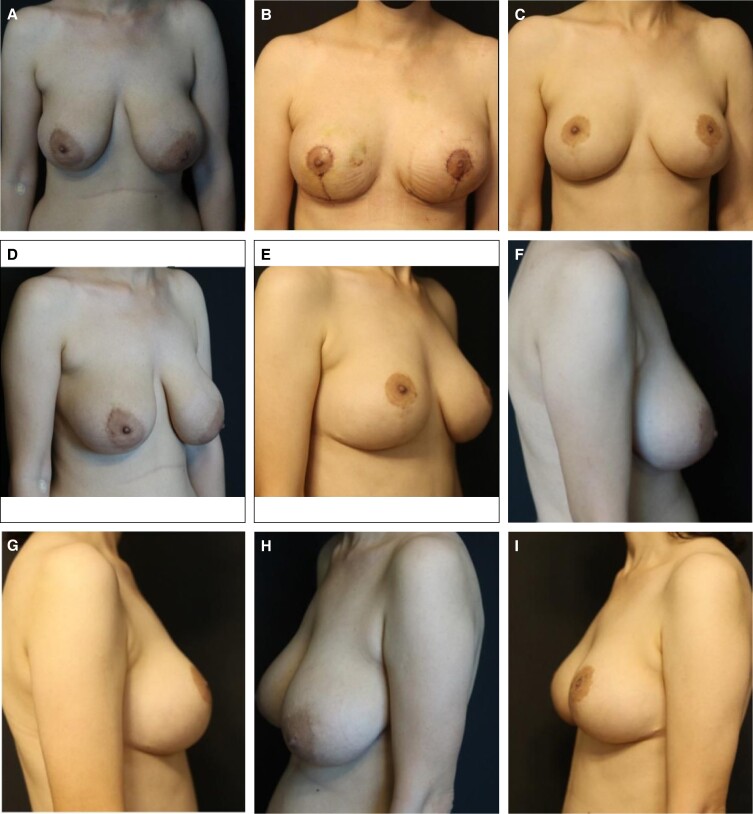
Photographs of a 38-year-old female patient who received autologous fat transfer to the breast. (A, D, F, H) before the surgery, (B) 1 week after the surgery, (C, E, G, I) 19 months after the surgery.

**Figure 2.**
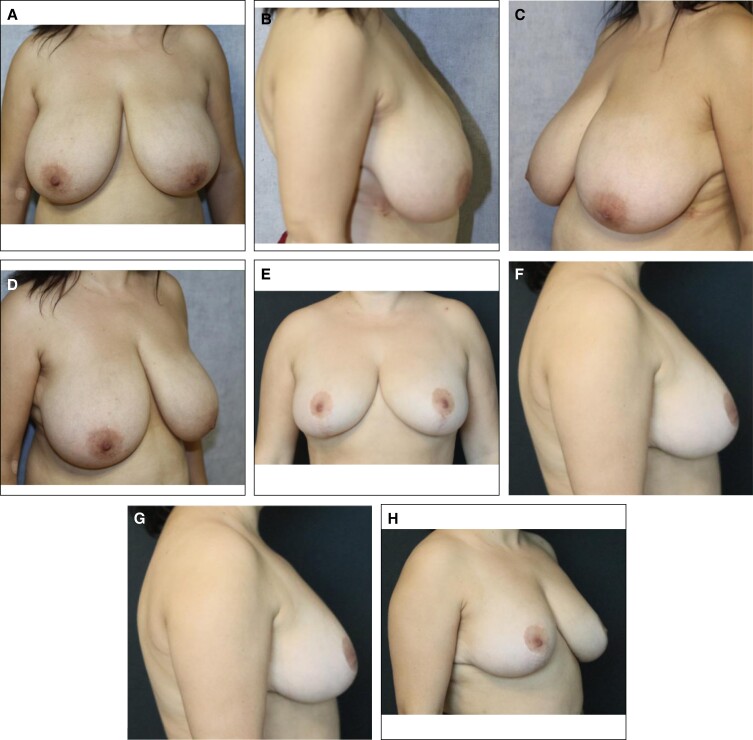
Photographs of a 34-year-old female patient who underwent reduction/augmentation-mastopexy procedure, and received autologous fat transfer to the breast. (A, C, E, G) before the surgery, (B, D, F, H) final result, 23 months after the surgery.

The preoperative and postoperative photographs of the patients are presented in Figures 1 and 2.

**Figure 3.**
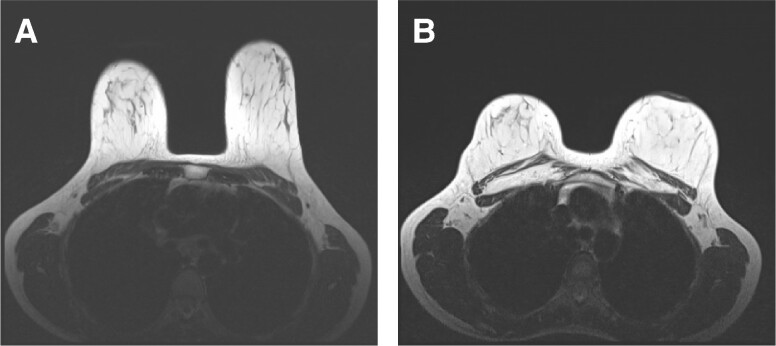
Exemplary MRI slices of the aforementioned patient. A 34-year-old female patient underwent a reduction/augmentation-mastopexy procedure, and received autologous fat transfer to the breast. (A) Before the surgery. (B) 19 months after the surgery.

The MRI scan evaluation of the fat resorption rate revealed a maintained breast volume and fat graft survival (Figure 3).

We did not encounter postoperative complications, and none of the patients underwent additional grafting procedures during the follow-up.

## DISCUSSION

Breast augmentation with implants is one of the most commonly performed aesthetic procedures worldwide. However, despite satisfactory results, it is associated with a revision and reoperation rate as high as 36% due to long-term complications such as capsular contracture, implant rupture or malposition, and ptosis.^10^ The correction and replacement procedures, including capsulectomy and capsulorrhaphy, are related to severe tissue trauma and atrophy, and high recurrence rates while revision procedures are challenging and may require the use of acellular dermal matrices, synthetic meshes, and autologous dermal grafts.^11-13^

In this study, we describe a VASER-assisted 4-layer AF transfer procedure to the breast, an approach that provides efficient and tailored breast contouring and improved graft survival. With this method, we utilize a natural aesthetic result based on the breast anatomy without the need for breast implants in patients suitable for the procedure.

In our study group, we reported a well-preserved breast volume, contour, and shape in female patients who underwent augmentation-mastopexy procedure, followed by autologous grafting of the fat. The sequential MRI evaluations on the follow-up visits demonstrated a symmetrical breast shape and sustained adipose tissue layer, corresponding to efficient graft survival.

The content of the fat grafts includes adipocytes, adipose tissue stem cells, preadipocytes, fibroblasts, vascular endothelial cells, and immune cells, whereas stromal and multipotent stem cell fractions are related to better graft survival, due to their capability of promoting angiogenesis around the injected autologous tissue.^14,15^ The fat absorption rate, an indicator of graft survival depends on the method and patient-related factors, such as the harvesting and lipoaspirate processing method, graft inoculation technique, as well as the breast thickness and volume, and only half of the grafted fat was expected to retain within the first year of the procedure.^16,17^ However, in our study, we observed an efficient adipose tissue survival, possibly as a consequence of the VASER-assisted harvesting procedure, which minimizes cellular injury to the adipocytes. We also applied a low suction vacuum during the harvesting, and the grafts were injected under low pressure with gentle movements.

In their experimental study, comparing the effects of traditional suction-assisted and ultrasound-assisted liposuction methods on fat graft survival, Hsiao et al reported histologically proven more angiogenesis and less fibrosis and apoptosis in the ultrasound group, whereas adipocyte stem cells showed a better differentiation capacity compared with the traditionally harvested tissue.^18^ The effect of the harvesting technique on graft survival has been reported in several experimental and clinical studies, and ultrasound-assisted liposuction to obtain the adipose tissue grafts is associated with higher cell viability and differentiation capacity.^6,19^ Moreover, significantly decreased levels of expression for inflammatory markers were revealed in another study, which were related to a lower antigraft response and higher tissue survival. In their study where they recruited varied energy settings for ultrasound devices, Genç et al showed reduced graft survival with higher ultrasonic energy levels, and they suggested an increased vibration amplitude over switching to a continuous mode for better outcomes.^20^

Although several studies suggested different techniques for breast augmentation with fat grafts, a standardization of the zones and areas to receive fat grafts has not been adopted by plastic surgeons.^21^ To the best of our knowledge, the present study is one of the limited numbers of studies, employing an MRI-based approach for the evaluation of fat graft survival in the follow-up of a homogeneous group of patients who underwent augmentation-mastopexy and 4-layer adipose tissue grafting.

In our technique, we injected the adipose tissue grafts under and into the pectoral muscle, under the pectoral fascia, and the subcutaneous layer in varying quantities. With this approach, we aimed to produce customized outcomes for each patient, based on the deformations, and shape and contour variations of each breast. Furthermore, we considered that a multiple-layer fat graft injection would provide a more efficient graft survival with the maintenance of the immediate postoperative results. We also avoided overinjection of fat grafts, and a limited amount of adipose tissue was administered to the patients for all layers.

In our study, we administered an approximate amount of 250 mL adipose tissue graft under and into the pectoralis muscle. The main blood supply to this muscle and surrounding tissues is obtained by the pectoral branch of the thoracoacromial artery; hence, the muscle is highly vascularized and well-perfused. Due to this reason, the pectoralis muscle is defined as the main pedicle for the musculocutaneous flap for several reconstructive procedures for its efficiency in tissue regeneration. Thus, we suggest that our technique allows for better survival of the adipose tissue grafts as a result of adequate vascularization of the recipient area.

On the contrary, estrogen was suggested as a promoting agent for angiogenesis and tissue vascularization, whereas elevated serum estrogen levels were shown to increase stem cell count and earlier vascularization of grafted fat tissue.^22^ Because the patient group presented herein is composed of nonmenopausal young females, a balance of estrogen hormone and its receptors in this group might contribute to the adipose graft survival. Moreover, our study group consisted of a well-chosen, uniform group of patients who would benefit from the procedure to a maximum level, and a thorough patient selection process in terms of muscle stiffness, tissue quality, and degree of disproportions is crucial for the best outcomes. In addition, AF grafts are convenient after breast cancer surgery and do not compromise the regular scans during the follow-up process.^23,24^ However, a detailed preoperative mammogram should be provided in order to determine the group of patients who could benefit from the procedure. For this group of patients, particular attention should be given to the differential diagnosis of calcifications in the grafts from tumor recurrence. It is worth mentioning that, in their research conducted across 17 breast units in Italy, Klinger et al assessed the outcomes of patients who underwent AF grafting, and compared the locoregional recurrence rate and locoregional recurrence-free survival between the groups. They reported that despite the suspected stem cell–like characteristics of adipocytes at the tumor site, AF grafting does not interfere with cancer prognosis.^25^

In our series, we did not observe previously defined recipient-site complications, including fat necrosis, oil cyst formation, indurations, and calcification in the MRI evaluations. However, we did not perform a tissue sampling study to confirm the findings, and this conclusion was based solely on the imaging modality. We also did not encounter decreased sensitivity, and severe deformity, and patients did not request an additional graft injection or prosthesis in the postoperative period.

Because a fat retention rate of 50% was expected within the first year following the procedure, MRI evaluations revealed a high survival rate of the transplanted adipose tissue at the end of the 18 months follow-up.

One of the major limitations of our study is the lack of a control group that encompasses nonultrasound-treated patients. However, all harvesting procedures for grafting are performed under ultrasound assistance in our practice. In addition, we did not perform an experimental study that histologically displays the outcomes in terms of apoptosis rate, vascularization, and tissue fibrosis, whereas we also did not perform a cellular study to evaluate the quality of the grafted fat. Given the difficulty of obtaining a biopsy specimen for patients who underwent breast augmentation with AF grafting, we concluded that an MRI scan would be an efficient tool to evaluate the fate of grafted material while assessing the patient for any graft-related long-term complications. However, MRI evaluations could not provide the amount of retained graft volume and the localizations for where the fat was retained and where it was lost. Moreover, the study's case count is limited, and all surgeries were performed by the same surgeon.

## CONCLUSIONS

Although the actual mechanism underlying long-term fat graft survival remains unclear, we conclude that a multiple-layer AF grafting is a safe and efficient procedure for the tailored sculpturing of the breasts in the augmentation-mastopexy surgery, regardless of the surgical technique performed.
